# Potential Importance of Maximal Upper Body Strength-Generating Qualities and Upper Body Strength Training for Performance of High-Intensity Running and Jumping Actions: A Scoping Review

**DOI:** 10.3390/sports12120357

**Published:** 2024-12-23

**Authors:** Ivan Curovic, David Grecic, David Rhodes, Jill Alexander, Damian J. Harper

**Affiliations:** 1 Institute of Coaching and Performance, School of Health, Social Work and Sport, University of Central Lancashire, Preston PR1 2HE, UK; jalexander3@uclan.ac.uk (J.A.); dharper5@uclan.ac.uk (D.J.H.); 2 Centre for Applied Sport, Physical Activity and Performance, University of Central Lancashire, Preston PR1 2HE, UK; dgrecic1@uclan.ac.uk; 3 Human Performance Department, D.C. United Football Club, Washington, DC 20003, USA

**Keywords:** sprinting, change of direction, repeated sprint ability, trunk muscles

## Abstract

Purpose: To investigate the influence of upper body (UB) strength qualities and UB strength training on the performance of high-intensity running and jumping actions and to identify gaps and recommendations for future research. Methods: A systematic search using the PRISMA Scoping Review protocol was conducted in February 2024 using PubMed, Scopus, and ICTRP. Studies eligible for inclusion were those that reported associations between UB or trunk maximal strength qualities (e.g., absolute strength, forces, power) and high-intensity running or jumping actions or investigated the influence of an isolated UB strength training intervention on high-intensity running or jumping performances. Results: Of the 4730 articles, 7 studies met the inclusion criteria, reporting correlations for 16 high-intensity running or jumping tests. No intervention studies were identified. Preliminary findings of the limited number of studies highlight that greater UB maximal strength-generating capacity may positively influence repeated sprint ability. While a significant moderate correlation between greater absolute UB strength and faster “flying” sprint was also reported, mixed results were found for sprint acceleration. There is also evidence that change-of-direction performance may greatly benefit from high maximal isometric strength of all trunk muscles and that strong trunk extensors may enhance drop jumps. Conclusions: This review identifies the potential of UB strength to contribute to high-intensity running and jumping actions. Future research is warranted to investigate this link via various UB strength tests and UB strength training protocols aimed at maximising neuromuscular adaptations.

## 1. Introduction

Many multi-directional sports are characterised by the necessity to perform frequent high-intensity running and jumping actions in which lower body (LB) muscles are often the main drivers of whole-body movements [1]. These actions involve sprinting across various distances, change-of-direction (COD), and jumping, which are often recognised as key performance components in sports such as soccer [1,2,3,4,5,6], rugby [7], basketball [8,9], and handball [8,10]. It has been widely demonstrated that LB muscle strength positively transfers to improved performance of these actions [11,12,13,14], with the ability to exert high forces during time-constrained tasks (i.e., impulse) being an important factor for maximising quick whole-body displacements [15,16].

Research on upper body (UB) muscle strength contribution to high-intensity running and jumping actions is limited to the exploration of coordinated exercises that involve both UB and LB segments, such as weightlifting [17,18], which makes it unclear whether strengthening of UB muscles independently of lower limbs could contribute to these actions. In sports with a high demand for tackles and physical duels, such as rugby [19,20], Australian Rules Football [21], and handball [22], UB strength and UB power have been reported to discriminate between players competing at different standards. However, these findings most likely exist due to the close-contact physical nature of those sports and not due to the improved high-intensity running and jumping potential. Importantly, any noted contribution of enhanced UB strength-generating qualities (e.g., absolute strength, peak forces, power) on isolated sprinting or jumping efforts may hold greater significance for the contextual nature of these actions during their execution in multidirectional sports. This could be especially relevant for sports such as soccer, where players do not seem to experience significant neuromuscular adaptations from UB resistance training sessions [23,24]. These athletes could, therefore, benefit from an increased UB activation in strength training plans [25], where augmented UB strength could be attained via only one exercise session per week [26].

It is possible that increased UB strength could make a positive contribution to high-intensity running and jumping actions by helping to increase the magnitude and duration of forces applied to the ground for acceleration and deceleration [27,28,29] and by improving force transmission to the lower limbs and trunk extensors by arm swings during jumping [30,31,32,33]. Another potential contribution to powerful LB contractions may be obtained via positive adaptations of the central nervous system (CNS) as a result of UB strength training sessions [34,35]. These adaptations could translate to improved rates of force development (RFD) [36], leading to enhanced performance on account of intensified neural drive [37,38]. Indeed, RFD measured by maximal handgrip has been associated with better jumping performance [39,40] and agility [41] in untrained recreational populations, although no such investigations appear to exist with athletes. The relationships between seemingly unrelated muscle groups likely exist as a result of central neural stimulations by strength or power training [35], which is supported by research reporting significant strength improvements in untrained upper extremities after leg exercise programmes [42,43] and successful post-activation potentiation for horizontal jumping following one-repetition maximum (1-RM) bench press [38]. Furthermore, UB resistance exercise may augment leg strength training responses via systemic anabolic influence [44,45] and protect LB muscle fibres from a catabolic environment after the leg-damaging sessions [46,47]. This could lead to the preservation of power-generating capacity in the lower extremities [47].

Unfortunately, the contribution of UB strength to high-intensity running and jumping actions has not gained much research attention, likely due to its lack of specificity to the nature of these movements. However, strength and conditioning coaches from elite soccer clubs expressed confidence in the role that UB strength and UB strength training could have for the enhancement of these actions [48]. Notably, systematic reviews with meta-analyses [49,50] reported only small effects of trunk muscle strength (i.e., “core muscles”) on the measures of athletic performance, although most of the included studies used core stability endurance tests and training exercises involving sub-maximal intensities (e.g., plank holds, double leg lowering) [49,50]. Testing and training of maximal trunk strength, however, might provide a more appropriate assessment of the role that these muscles could have for powerful movements of the whole body in the athletic context [51]. Maximal strength is defined as the ability of the muscles to exert maximal forces against high resistance [52,53], which is associated with improved athletic performance in multidirectional speed sports [54,55]. In addition, it may be important to distinguish between the strength of trunk flexors and trunk extensors because only the latter muscle group shows promising results with its contribution [51,56]. Therefore, the aim of this scoping review is to explore the importance of maximal UB strength-generating qualities (inclusive of trunk muscles) and UB strength training for the performance of high-intensity running and jumping actions. A further aim is to identify gaps in current research with a view to recommending future research directions.

## 2. Methods

### 2.1. Experimental Approach to the Problem

Scoping reviews have been conducted to map and discuss newly emerging concepts within a selected research area [57]. Unlike standard systematic reviews that address more specific questions with a clear availability of relevant studies, scoping reviews are used to examine emerging areas of research with the clarification of key concepts [58]. This permits the examination of a broad range of literature, with the goal of the current review being to identify potential links between UB strength and high-intensity running and jumping performances. Utilising the PICO framework for guidance [59], the focus was placed on identifying the importance of UB strength-generating qualities (cross-sectional studies) and UB strength training (intervention studies) on the performance of high-intensity running and jumping actions in trained males or females between the ages of 16 and 35 years. Therefore, the outcomes investigated in this review were linear sprinting (including acceleration and maximum velocity), COD (including deceleration performance), jumping (including squat jump, countermovement jump (CMJ), and drop jump variations). Additionally, we included repeated sprint ability (RSA) considering that this activity involves repeated short-duration sprints and/or CODs and is highly applicable to multidirectional sports [60]. The UB segment investigated in this review included the upper torso area (involving back muscles, chest, shoulders, and upper limbs) and trunk muscles (involving trunk flexors, lateral flexors, and trunk extensors). Thus, any strength test result assessing trunk muscles will be specifically referred to as “trunk strength”, while all other UB test results (e.g., bench press, lat pull-down) will be referred to as “UB strength”.

### 2.2. Search Strategy

This study adheres to the Preferred Reporting Items for Systematic Reviews and Meta-Analyses extension for Scoping Reviews (PRISMA-ScR) guidelines [61] following the method described by Moher et al. [62] to ensure comprehensive and transparent reporting throughout the process (Table 1). A systematic search of three electronic databases (PubMed, Scopus, and International Clinical Trials Registry Platform) was performed in February 2024 by the lead author (IC) using the default field search setting within each database. Only original peer-reviewed articles written in English were considered. The terms that were searched in titles, abstracts, and keywords were “upper body strength” OR “upper body training” OR “trunk strength” AND “high intensity actions” OR “high-intensity actions” OR “sprint” OR “jump” OR “change of direction”. In addition, the reference lists of all included studies were screened.

### 2.3. Study Selection Criteria

To address the potential scarcity of studies examining associations and interventions involving leg-independent maximal UB strength-generating qualities and high-intensity running and jumping actions, the search process was designed to ensure comprehensive coverage of the three databases added by citation chasing and grey literature exploration (reported as “other sources”) [63]. Two authors independently screened abstracts and full texts based on explicitly defined inclusion and exclusion criteria to accurately identify eligible studies focusing on UB or trunk strength tests and interventions related to high-intensity running and jumping actions. Any discrepancies were resolved through discussion (*n* = 4). Studies eligible for inclusion were those that (1) reported associations between the measures of maximal UB or trunk strength-generating qualities (e.g., absolute strength, forces, power) and maximal sprinting, COD, or jumping performance, (2) investigated the effect of an isolated (i.e., leg-independent) UB strength training intervention effect on the performance of sprinting, COD, or jumping, and (3) tested or trained males or females between the ages of 16 and 35 years to achieve greater relevance for athletic population [64,65]. Studies were excluded from the review if they (1) tested muscular endurance instead of neuromuscular strength qualities [52,53,66] and (2) implemented a training intervention focused exclusively on trunk musculature.

### 2.4. Data Extraction

All data for each study were extracted by the lead author (IC), including (1) study details (author and year of publication), (2) participant characteristics (sample size, population, sex, age, body mass, and height), (3) UB tests used, (4) HIA tests used, (5) associations between UB and LB tests, and (6) key findings. Following initial data extraction, a random subset of studies was assigned to another author (DH) to check the accuracy of the extracted data. This approach ensured the reliability of the collected information, minimising errors or discrepancies in the dataset. The magnitude of correlations reported between UB strength and HIA were interpreted using the scale from Hopkins [67] as trivial (*r* = 0.0–0.09), small (*r* = 0.10–0.29), moderate (*r* = 0.30–0.49), large (*r* = 0.50–0.69), very large (*r* = 0.70–0.89), nearly perfect (*r* = 0.90–0.99), and perfect (*r* = 1.00).

## 3. Results

### 3.1. Search Results

**Figure 1:**
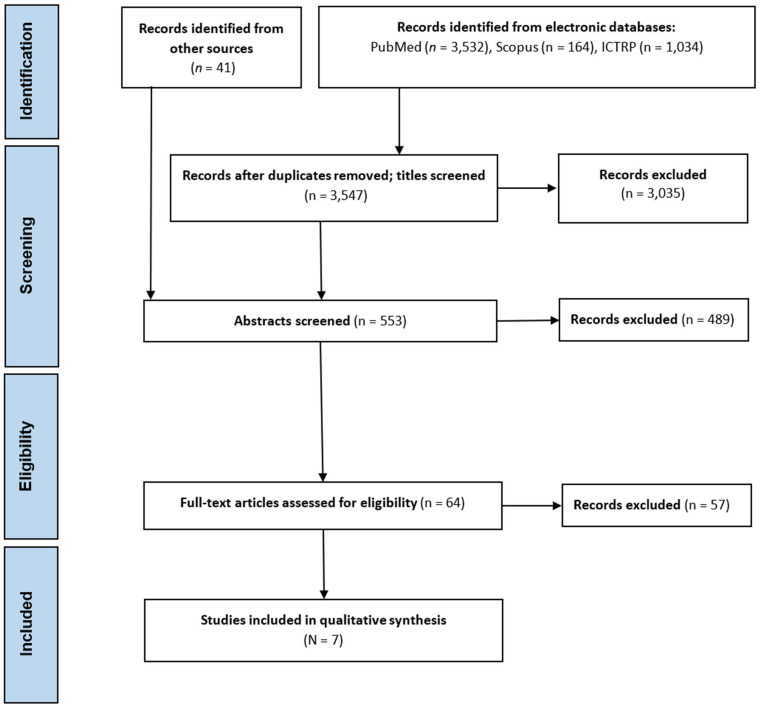
PRISMA flowchart illustrating step by step process leading to the identification of studies eligible for the scoping review.

Initial database searches resulted in the identification of 4730 articles. Following the removal of duplicates (*n* = 1183) and titles screened (*n* = 3035), 512 remaining studies were joined by articles identified through other sources (*n* = 41), resulting in 553 records subjected to abstract screening (Figure 1). Subsequently, 489 articles were excluded for not meeting the inclusion criteria, leaving 64 articles eligible for full-text screening. Finally, after removing 57 articles for various reasons (e.g., the absence of isolated UB strength tests, irrelevant running tests), 7 articles remained for final inclusion. There were no UB strength training intervention studies identified to be eligible for this review.

### 3.2. Characteristics of Included Studies

Two studies had mixed-sex participants [68,69], four studies had only males [70,71,72,73], and one had only females [74]. The age range of participants was 16–24 years inclusive of all eligible studies. The outcomes included sprinting tests (*n* = 6) [71,72,73], RSA tests (*n* = 4) [70,71,73], COD tests (*n* = 2) [74], and jumping tests (*n* = 2) [68,69] via vertical CMJ (*n* = 1) [69] and drop jump (*n* = 1) [68]. UB strength was tested with bench press (*n* = 5) [69,70,71,72,73], shoulder press (*n* = 1) [73], and lat pull-down [73], while trunk strength was tested via maximal torque of trunk extensors (*n* = 2) [68,74], flexors (*n* = 2) [68,74], and lateral flexors (*n* = 1) [74]. Six studies [69,70,71,72,73,74] involved various athletic populations, including Australian Rules Football (*n* = 1) [72], basketball (*n* = 3) [69,70,74], handball (*n* = 1) [71], and soccer (*n* = 1) [73]. One study involved a trained recreational population, examining drop jumping performance [68]. Sample sizes varied between 11 and 327 participants across all studies. Amongst the five studies that investigated UB strength [69,70,71,72,73], the sample size was between 11 [70] and 34 [72], with population groups including elite male athletes (*n* = 45) [70,72], collegiate mixed-sex athletes (*n* = 13 for males, *n* = 12 for females) [69], and male youth athletes (*n* = 51) [71,73]. Of the two studies [68,74] investigating trunk strength, the sample size was 29 for trained males (*n* = 14) and females (*n* = 13) [68] and 327 for various male and female athletes [74].

### 3.3. Correlations Between Upper Body Strength and High-Intensity Actions

Table 1 provides a summary of the characteristics, outcome measures, results, and key findings of the seven cross-sectional studies that investigated correlations between UB strength and high-intensity running and jumping performances. Five of these studies tested maximal UB strength [69,70,71,72,73], while two studies tested maximal trunk muscle strength [68,74].

**Table 1:** Summary of cross-sectional studies investigating correlations between upper body strength and performance of high-intensity running and jumping actions.

Study	Participant Description	Upper Body Strength Test	High-Intensity Action Test	Results	Key Findings
Young et al. (2005) [72]	34 elite male AFL players (age 22.7 ± 3.4; stature 187 ± 8 cm); body mass 88.0 ± 8.9 kg	- 3 RM bench press (kg)	- 10 m and 30 m sprint (s) - Flying 30 m sprint (s)	- 3 RM bench press and flying 30 m sprint (*r* = −0.41, *p* < 0.05) - No significant correlations between other sprint times and UB strength	Greater maximal UB strength positively influenced maximal speed performance.
Walsh et al. (2007) [69]	13 male collegiate basketball players (age 19.7 ± 1.1; stature 195.8 ± 10.4 cm; body mass 97.1 ± 15.3 kg) 12 female collegiate basketball players (age 19.1 ± 1.2; stature 179.4 ± 8.8 cm; body mass 74.4 ± 9.2 kg)	- 1 RM bench press/kg	- Vertical SJNA (cm) - Vertical CMJNA (cm) - Vertical SJA (cm) - Vertical CMJA (cm)	- 1 RM bench press and contribution of arm swing to CMJA height for men (*r* = 0.27; *p* < 0.05) and women (*r* = −0.12; Small, *p* < 0.05)	Greater maximal relative UB strength did not have a significant influence on arm-swing contribution to jumping performance.
Ingebrigtsen & Jeffrey (2012) [71]	29 elite male youth handball players (age 16.5 ± 0.8; stature 184.3 ± 4.8; body mass 77.0 ± 9.4 kg)	- 1 RM bench press/kg	- 10 m and 30 m sprint (s) - 10 m and 30 m RSA (6×) (s)	- 1 RM bench press and 10 m RSA times (*r* = −0.56, *p* < 0.05, and *r* = −0.57, *p* < 0.01, for absolute and relative strength, respectively)	Greater maximal and relative UB strength positively influenced 10 m RSA performance.
Balsalobre-Fernández et al. (2014) [70]	11 elite male basketball players (age 24.5 ± 5.8; stature 200 ± 10.9 cm; body mass 98.4 ± 9.0 kg)	- Four concentric-only reps on a Smith bench press with 47.5 kg, 57.5 kg, 67.5 kg, and 77.5 kg loads with peak power (W) and force (N) measured at each load - 1 RM bench press (kg)	- RSA (6 × 35 m) with sprint power (weight × distance^2^)/time^3^ (W), fatigue index (RSA maximum/minimum power)/RSA maximum power × 100 - CMJA height (cm) before and after RSA	- 1 RM bench press and RSA fatigue index (*r* = −0.61, *p* < 0.05) - Bench press peak power and RSA fatigue index (*r* = −0.74, *p* < 0.05) - Bench press peak power and CMJA height loss (*r* = −0.77, *p* < 0.01) - Bench press peak force and RSA fatigue index (*r* = −0.86, *p* < 0.01) - Bench press peak force and CMJA height loss (*r* = −0.84, *p* < 0.01 for 77.5 and 67.5 kg loads; *r* = −0.79, *p* < 0.01 for 47.5 kg load)	Greater maximal UB strength and concentric UB force and power positively influenced RSA performance.
Tunçel et al. (2023) [73]	22 young male soccer players (age 16.20 ± 0.77; stature 174.7 ± 5.1 cm; body mass 68.0 ± 10.0 kg)	- 1 RM bench press (kg), shoulder press (kg), and lat pull-down (kg)	- 10 m and 30 m sprint (s) - Bongsbo’s 7 × 35 m repeated (COD) sprint ability test	- 1 RM shoulder press and 10 m, 30 m sprint, and repeated (COD) 30 m sprint ability times (*r* = −0.502, *r* = −0.598, *r* = −0.485, respectively, *p* < 0.05)	Greater maximal UB (shoulder) strength positively influenced short sprinting and RSA performance.
Prieske et al. (2015) [68]	14 trained males (age 22.9 ± 2.4; stature 179.9 ± 6.1 cm; body mass 74.4 ± 8.0 kg) 15 trained females (age 23.8 ± 3.3; stature 169.6 ± 8.9; body mass 60.3 ± 8.4 kg)	- Peak isokinetic torque of trunk extensors and flexors (Nm)	- DJ height (cm), DJ performance index (ratio between the DJ height and CT)	- Peak isokinetic torque of the trunk extensors (but not flexors) and DJ height (*r* = 0.64 for stable surface, *r* = 0.66 for unstable surface, *p* < 0.01) - Peak isokinetic torque of the trunk extensors (but not flexors) DJ performance index (*r* = 0.50 for stable surface, *r* = 0.60 for unstable surface, *p* < 0.01)	Greater trunk extensor (but not flexor) strength positively influenced drop jump performance.
Kozinc et al. (2021) [74]	327 male and female athletes: basketball players, tennis players, runners (age 18.6 ± 8.1; stature 179.2 ± 10.5; body mass 71.2 ± 13.1 kg)	- Maximal isometric torque of trunk extensors, flexors, and lateral flexors (Nm/kg)	- COD 90° and COD 180° (s)	- Isometric trunk extensor strength and COD 90°/COD 180° times for basketball players (*r* = −0.42 and *r* = −0.36, respectively, *p* < 0.01), runners (*r* = −0.62 and *r* = −0.36, respectively, *p* < 0.01), and tennis players (*r* = −0.39 and *r* = −0.49, respectively, *p* < 0.01) - Isometric trunk flexor strength and COD 90°/COD 180° times for basketball players (*r* = −0.39 and *r* = −0.32, respectively, *p* < 0.01), runners (*r* = −0.68 and *r* = −0.02, respectively, *p* < 0.01), and tennis players (*r* = −0.48 and *r* = −0.61, respectively, *p* < 0.01) - Isometric trunk lateral flexor strength and COD 90°/COD 180° times for basketball players (*r* = −0.42 and *r* = −0.38, respectively, *p* < 0.01), runners (*r* = −0.67 and *r* = −0.02, respectively, *p* < 0.01), and tennis players (*r* = −0.47 and *r* = −0.56, respectively, *p* < 0.01)	Greater trunk extensor, flexor, and lateral flexor strength positively influenced COD performance.

AFL = Australian Football League, CMJNA = countermovement jump with restricted arms, CMJA = countermovement jump with free arms, COD = change of direction, CT = contact time, DJ = drop jump, N = newtons, RM = repetition maximum, RSA = repeated sprint ability, SJA = squat jump with free arms, SJNA = squat jump with restricted arms, W = watts.

Regarding the measures of UB strength and sprinting, there were significant large negative correlations between maximal shoulder press and 10 m and 30 m sprint times (*r* = −0.502 and *r* = −0.598, respectively, *p* < 0.05) [73] and significant moderate negative correlations between maximal bench press and “flying” 30 m sprint times (*r* = −0.41, *p* < 0.05) [72], indicating positive influence of greater UB pushing strength on short sprint performance and maximal sprinting speed [72,73]. However, other study findings showed that 10 m and 30 m times were not significantly affected by other UB strength assessments measured by bench press [71,72,73] and lat pull-down [73]. Furthermore, significant moderate negative correlations were reported between maximal shoulder press and 30 m RSA times for young soccer players (*r* = −0.485, *p* < 0.05) [73], while significant large negative correlations were reported between maximal bench press and 10 m RSA times for young handball players (*r* = −0.56, *p* < 0.05, and *r* = −0.57, *p* < 0.01, for absolute and relative strength, respectively) [71], and 35 m RSA fatigue index in professional basketball players (*r* = −0.61, *p* < 0.05) [70]. In addition, Balsalobre–Fernández et al. [70] reported very large negative correlations between the calculated peak power and peak force from bench press and two outcomes related to fatigue emergence, including RSA fatigue index (*r* = −0.74, *p* < 0.05, and *r* = −0.86, *p* < 0.01, for peak power and peak force, respectively), and CMJ height loss in the second series of jumps (*r* = −0.77, *p* < 0.01, and *r* = −0.84, *p* < 0.01 for 77.5 and 67.5 kg loads; *r* = −0.79, *p* < 0.01 for 47.5 kg load, for peak power and peak force, respectively). These findings suggested a positive influence of greater UB strength on RSA performances. Lastly, despite the significantly higher contribution of arm swings to vertical CMJ height in men compared to women [69,75], the influence of 1 RM bench press on the arm-swing effect toward vertical jumping was unclear [69].

Regarding the measures of trunk strength, Prieske et al. [68] reported significant large positive correlations between the peak isokinetic torque of trunk extensors and drop jump height (*r* = 0.64 for stable surface, *r* = 0.66 for unstable surface, *p* < 0.01), as well as moderate-to-large correlations with performance index (*r* = 0.50 for stable surface, *r* = 0.60 for unstable surface, *p* < 0.01). Notably, trunk flexor strength was not significantly associated with the jumping performance in this study [68]. Another study reported moderate-to-large significant correlations between maximal isometric torque of all trunk muscles (i.e., trunk extensors, flexors, and lateral flexors) and both 90° and 180° COD performances for basketball players, tennis players, and runners [74].

## 4. Discussion

The aim of this scoping review was to explore the importance of maximal UB strength-generating qualities (e.g., absolute strength, forces, power) and UB strength training for the performance of high-intensity running and jumping actions. A further aim was to identify gaps in current research with a view to recommending future research directions. The main findings highlight that greater maximal strength and force-generating capacity of UB muscles may positively influence RSA. While a positive influence of absolute UB strength toward maximal “flying” sprint is also reported, mixed results have been found for sprint acceleration. Furthermore, there is evidence that COD performance may benefit from greater maximal isometric strength of all trunk muscles and that strong trunk extensors may enhance drop jumps, showing promise for enhancing other jumping variations. Further research is necessary to substantiate these findings and investigate the influence of different UB strength qualities and UB strength training protocols on high-intensity running and jumping performances.

### 4.1. Upper Body Strength and Sprinting Performance

This review highlights some mixed findings in relation to the importance of UB strength for sprinting performance. In elite Australian Rules football players, Young et al. [72] reported that greater absolute UB bench press strength was moderately supportive for maximal sprinting speed measured by 30 m flying times. However, 10 m and 30 m sprint distances were not significantly influenced by maximal bench press strength [71,72,73], although one study reported large significant correlations with maximal shoulder press strength [73].

A possible explanation for enhanced maximal sprinting speed performance (i.e., 30 m flying time) with greater unspecific strength of UB muscles could be due to the higher ground reaction forces (GRF) produced by stronger athletes. For example, Weyand and Davis [76] found that higher whole-body muscle mass exhibited a greater magnitude of GRF that directly contributed to the sprinting speed of elite runners. Other studies have affirmed that a top running speed is dominantly achieved through the superior vertical components of GRF applied over a short period of time [27,28], which could be supported by the greater muscularity (i.e., strength) of the UB region [77]. Moreover, stronger athletes consistently show increased peak RFD [36,78], likely via improved central motor outputs for well-trained individuals [36]. Indeed, one of the crucial neuromuscular adaptations to strength training involves intensified central neural drive [79,80,81], which may lead to augmented force production for maximal sprinting [37]. The central neural drive could be influenced by the strength training of unspecific muscle groups to the desired action [35,38]. For example, leg resistance training programmes have been shown to reliably lead to the increased strength of untrained upper extremities in the recreational population [42,43], demonstrating neurological benefits for the muscles that were not even active during the exercise period. Hence, UB strength training might be partially responsible for the potentiated RFD toward the upper and lower limbs, offering a sensible explanation for the positive link reported between unspecific UB muscles and sprinting performances [72,73]. Another potential contribution of UB strength may arise from the biomechanical advantage obtained by energetic upper limb movements. While arm drive does not seem to directly contribute to the horizontal propulsion of the body due to the synchronised cancelling arm movements in the opposite directions [82], the vertical component of arm swing increases the total body lift [82], doing so significantly more in sprinting (27%) than in distance running (7%) [83]. This helps the sprinters stay off the ground for longer, thus allowing them to apply greater GRF and move faster [27,28,76]. Therefore, UB strength needs to be further evaluated with its potential to positively contribute to maximal sprinting speed.

The unclear link between UB strength and short sprinting performances (<30 m) could be due to several factors. For example, it is possible that LB strength levels and positional requirements differed between the tested athletes who competed in handball [71] and Australian Rules Football [72]. If the overall body mass is not supported by high relative strength capacity, short sprinting performance will likely be worsened [84,85]. Unfunctional weight (i.e., fat mass) incapable of contributing to rapid muscle contractions could only be a burden for sprinting [77]. It is also important to note that the ability to generate high peak force is not necessarily associated with the ability to rapidly activate the muscle [16], especially when this muscle does not directly contribute to the performance in question [86]. For example, if a playing position requires more physical duels and fewer running tasks [87], it may be the case that the greater UB pressing strength of the tested players was not relevant enough for short sprinting requirements [72]. Moreover, several researchers consider arm actions to be only a passive natural movement during running [88,89], with their primary role believed to be a counterbalancing angular momentum of the lower extremities [90]. Brooks et al. [91] supported this argument, demonstrating that restricted arm motion compromised 20 m sprint times by less than 0.10 s. Acknowledging LB muscles as primary movers in sprinting, authors suggested that compensatory mechanisms with more significant upper torso rotations generated by other UB muscles were able to offset arm movement isolation [91]. Accordingly, high-velocity body displacements in the sagittal and transverse planes do not seem to be assisted by arm motion alone but, arguably, by joint forces generated in other UB areas, requiring the activation of diverse muscle groups. Unfortunately, maximal bench assessments were predominantly used to identify associations between UB strength and sprint performances in this review. It is unclear what type of strength tests could reflect rapid arm and trunk movements, but it is likely that bench press will have little specificity toward these patterns [92]. For example, Hinrichs [93] reported 40–60% of maximal voluntary isometric contraction for various UB muscles during the submaximal running speeds, with latissimus dorsi and posterior deltoid showing the highest activation. These two muscles are primarily trained via different pulling movements [94,95], which is not reflective of the pushing strength that bench press exercise reveals [92].

Acceleration performance (i.e., 10 m sprints) was significantly correlated with UB strength in one study [73], while two reported non-significant correlations [71,72], which may be explained by the variance in body mass as an influencing factor. For example, there may be a ceiling effect beyond which greater UB strength may fail to enhance short sprinting actions due to excessive hypertrophy, which could increase the momentum demands associated with larger muscles in this segment [54]. Unlike the maximal velocity phase, acceleration requires longer ground contact in which explosive concentric strength of LB muscles produces horizontally oriented forces to support momentum in the wanted direction [96]. Therefore, minimising body weight looks critical for achieving faster acceleration [97], and only in this way would the ability to manipulate the UB segment and generate high RFD become relevant for supporting legs with accelerative capabilities [98]. Notably, the study that demonstrated the positive influence of maximal UB strength on acceleration performance tested soccer players with leaner statures [73] compared to two studies that tested handball and Australian Football athletes with higher body weight [71,72], which might have influenced the results. With a forward-orientated UB required for sprint starts, the vertical force component gained by arm drive becomes important for horizontal propulsion due to the forward shift of the body’s centre of mass [96,99,100,101]. Scapular and humerothoracic joints have been shown to contribute to greater upper- and lower-extremity motions in this phase [102,103,104]. More research is, therefore, required to unveil potential associations between relative UB strength qualities and short sprinting performance [97] that appear relevant for multidirectional sport athletes [8].

Although no studies were identified that investigated the influence of trunk muscle strength on sprinting, Kubo et al. [56] highlighted the importance of trunk extensors (i.e., erector spinae and quadratus lumborum) for acceleration due to the significant positive associations observed between the size of these muscles and short (<20 m) sprinting tasks. Somewhat surprisingly, the same link was not found for trunk flexors [56]. Similarly, Ben Moussa Zouita et al. [51] revealed that only the strength of trunk extensors effectively separated high-level athletes and recreational populations, whereas no such associations were found for trunk flexors. Future research is needed to investigate how different UB strength tests, with a particular emphasis on those involving back extension, and different UB strength qualities (e.g., fast dynamic, heavy dynamic, reactive, maximal isometric, and explosive) are associated with sprinting [105]. It is also important to understand how these qualities may differ in importance for different sprint phases (i.e., acceleration and maximal velocity) that have unique kinetic and kinematic requirements [86]. Additionally, exercise interventions should prioritise dynamic strength exercises that emphasise strengthening UB muscles, with a particular focus on back extension movements [106].

### 4.2. Upper Body Strength and Repeated Sprint Ability

While no studies examining the relationship between trunk muscles and RSA have been identified, an interesting and potentially important finding from this review showed that greater UB strength was associated with improved RSA [70,71,73]. It may be speculated that the repeated force production in LB was supported by the more effective use of UB muscles for these actions [69,82,83,93], which would lower the fatigue occurrence in players with a stronger UB segment. Another explanation may arise from the neurological advantage that the athletes acquired with improved neuromuscular qualities. Stronger individuals could be more resistant to the development of central fatigue with repetitive maximal and submaximal efforts due to improved motor cortex outputs with less neural cost for the same voluntary activations [52]. There appears to be a strong link between the ability to produce maximal contractions during the initial phase of explosive movements and RFD [36], which also reaches peak levels during the acceleration phase of sprinting [36]. In accordance, Balsalobre–Fernández et al. [70] reported that UB concentric peak force and power were significantly associated with better RSA fatigue resistance. Therefore, persistent strength training of UB muscles might have resulted in favorable alterations of the CNS capability to increase neural impulses to the motoneuron pool and muscle fibers, thereby reducing the supraspinal fatigue occurrence [34] in repeated short sprints [70,71,73]. It is important to highlight that a higher fat mass will, logically, have a negative impact on RSA because of the increased energy demands during the sprinting efforts [107]. Even the ratio of muscle mass to its strength-generating capacity could be a compromising factor, further highlighting the importance of neuromuscular (relative) strength performance [54]. For instance, absolute maximal bench presses were negatively associated with anaerobic shuttle run tests only when the results were not adjusted to the body weight of the participants [108]. In contrast, UB strength–endurance successfully predicted players’ resistance to fatigue [74]. Therefore, it is sensible to suggest that UB strength should be expressed relative to body mass whenever analysed with its contribution to repeated high-intensity actions, especially when these actions require longer intermittent sprinting times with high-intensity decelerations involved [73,109].

### 4.3. Upper Body Strength and Jumping Performance

The findings of the current review highlight an unclear association between UB strength and jumping performance [69] and suggest trunk muscle extensors (i.e., erector spinae muscle group) as potentially important contributors to drop jumps [68]. Two studies [69,75] reported a higher contribution of arm swing to the jumping height in male basketball players compared to females, despite unclear associations to 1 RM bench press [69]. This positive arm swing contribution could be due to the enhanced GRF and takeoff velocity [110,111] as a result of arm swing-augmented force production for legs in the propulsive phase [30,31,112] and imparted energy to the system generated by the work of shoulder muscles [110,113]. Those mechanisms explain that arms are able to “pull” the trunk upward at takeoff with the net forces arising from shoulders and elbows [110,113]. This might be especially noted in male athletes and younger populations for whom arm swing initiates a proximal-to-distal jumping technique [114,115], in which the order of joint extensions starts from the hip, followed by the knee and ankle joints [116], resulting in the avoidance of antagonist co-contraction in the upper leg muscles and enhancement of performance [117]. Indeed, the importance of additional work by shoulder joints in arm-swing jumps [113,118,119] is reflected through the augmentation of forces produced around the hip joint, which is one of the key mechanisms contributing to improved jumping results [113,117,120,121]. It is, therefore, likely that the reported greater arm-swing enhancement of vertical jump for men compared to women was made possible via extended force production around the hip joint by the muscles whose strength cannot be revealed with bench press [69,75].

Importantly, hip extensors are not the only muscle group that generates high forces in this area. Blache and Monteil [119] added the pelvis to a musculoskeletal system simulation model applied to jumping participants and found that the erector spinae muscles (i.e., trunk extensors) accounted for 66% of the total work increase in arm-swing protocols, possibly due to the increased forward lean of the trunk gained by arm movements that allowed for a greater displacement of the body’s centre of mass and more emphasised trunk extension. Moreover, there are detriments in jump height when this group of muscles is fatigued beforehand [122] or when its activation is limited with an erected spine before the jump [123,124]. In accordance, Prieske et al. [68] reported significant large positive correlations between the trunk extensor strength and drop jump height, while no such associations were found for trunk flexors. Therefore, while bench press may not be the most valid UB strength test to reflect this ability, trunk extensors could be a suitable target for further investigations concerning sports with repeated jumping requirements [125], along with examining associations with other UB strength qualities, as highlighted in the previous section. Notably, trunk training interventions that targeted trunk strength-endurance have reported conflicting results toward jumping actions [126,127], while a dynamic trunk exercise programme improved both vertical and horizontal jumping performance in junior soccer players significantly more than static “core” training [106].

Other UB strength qualities, such as peak RFD, may be relevant for the augmentation of force transfer from the upper extremities to the lower limbs [118,120]. Da Silva et al. [38] revealed that neuromuscular potentiation obtained by UB pre-conditioning activity with heavy bench pressing successfully augmented a horizontal jumping task in female soccer players, suggesting the CNS role for high-intensity contractions in unrelated body areas. This shared effect may become important for jump improvements on account of intensified neural drive by the strength training of unspecific muscle groups [34,35] via its link to increased RFD [36]. Indeed, recreational individuals show a reliable positive association between the RFD measured by the handgrip strength test and jumping performances [39,40]. This unspecific link to UB strength was also made plausible by Iguchi et al. [128], who recorded the highest CMJ for the group of athletes with the greatest relative 1 RM bench press despite not being the strongest with the relative 1 RM squat. Unfortunately, the authors did not report correlations between the tested groups, so the conclusion for UB strength contribution remains speculative and likely of only a small effect. In summary, more research is warranted, and future investigations should involve relative UB force calculations accompanied by body composition analysis to allow for the consideration of fat tissue mass, which has been demonstrated to have adverse effects on jumping actions [84,85,129]. Furthermore, UB strength exercise regimes aimed at maximising neuromuscular adaptations are warranted for further exploration, emphasising fast downward braking capabilities of a trunk via eccentric contractions of back muscles and quick, powerful transition to the concentric phase via trunk extension after landing [68].

### 4.4. Upper Body Strength and Change-of-Direction Performance

There were no studies identified in this review that investigated the influence of maximal UB strength-generating qualities on COD performance. That said, maximal strength of all trunk muscles was found to be significantly associated with improved COD performance times in two COD tests, with its impact surpassing even that of ankle and knee joint forces [74]. Trunk musculature might support COD movement patterns via more effective UB manipulations that contribute to higher impulse generation in separate sub-phases [109,130,131,132]. Leaned torso with rapid upper limb swings have been reported to contribute to all of the COD sub-phases, including acceleration [102,103,104], deceleration [29,133], turn [133], and re-acceleration [134]. This effect might be somewhat explained by Gracovetsky’s spinal engine theory [135], which proposes that the spine itself acts as a crucial engine in locomotion, coordinating with trunk movements for efficient pelvic control independent of leg influence. While the relevance of traditional static “core” exercise plans for enhancing these dynamic COD movements is questionable [126,127,136], dynamic trunk strength training has shown promise in improving COD performance among young (12–14 years) soccer players [106]. Furthermore, increased trunk strength obtained via neuromuscular adaptations [54] may contribute to improved RFD [137] that helps control trunk position to initiate momentum to the desired movement direction [36,133]. Similarly, trunk muscles’ ability to quickly decelerate before making a turn [132,133] may be realised via forceful contractions rapidly transforming from eccentric to isometric and concentric contraction phases [132]. This could explain not only the positive associations reported between the maximal isometric torque of all trunk muscles and two different COD tasks [74] but also the relationship between trunk extensors’ peak isokinetic force and drop jump height previously reported [68]. Therefore, future research should isolate and quantify the individual contributions of different trunk muscle groups to COD ability using different strength assessments in dedicated trunk flexion, rotation, and extension exercises.

It is currently unknown if higher levels of strength in other UB areas could serve as facilitators of force transmissions to the LB in COD patterns and whether focused UB strength training interventions may facilitate superior COD performances. One could argue, however, that the amount of relative strength, particularly in the arms and upper back, is contributive to quick turns and directional shifts with the role of maintaining balance and initiating momentum through trunk (re)positioning [138,139]. Moreover, UB strength exercise may intensify the central neural drive directed toward the lower limbs [38], which could provide assistance for whole-body movements [140]. For example, RFD measured by maximal handgrip served as a reliable predictor of agility in older populations [41], although no such investigations have been identified with athletes. Furthermore, Loturco et al. [141] showed that rugby and handball players were capable of running and changing direction significantly faster than soccer players, potentially suggesting that the greater UB strength needed in these sports [142,143] compared to soccer [26] might have positively affected COD efficiency. Therefore, to better understand how UB muscles may uniquely affect various sub-phases of COD, a dedicated separate analysis is required examining the influence of diverse UB strength qualities (e.g., RFD, peak power, peak forces, eccentric, isometric, concentric) on each of the COD components. It also looks important for UB strength to be normalised to body mass in order to be discussed for its relevance toward these actions [55]. Consequently, thoughtful consideration around the approaches to UB strength improvement on account of neural adaptations [34,37] is warranted, factoring in the LB strength levels of the participants [55] for future interventions.

A primary limitation of this review is the paucity of studies that have investigated the influence of different UB strength measures on high-intensity running and jumping actions. UB strength was primarily tested with bench press using the maximal resistance that could be lifted, which prevented the evaluation of a more diverse range of strength qualities and muscle groups from this area. Furthermore, sample sizes varied between 11 and 327 participants across the included studies, which could inevitably influence the magnitude of the reported correlations. Additionally, the measures of leg strength and power were not accounted for when UB influence was analysed, yet these factors are likely to have a much more significant impact on all high-intensity actions investigated in this review. Notably, baseline UB strength levels could have been insufficient to significantly affect the outcomes which, along with the lack of relative strength calculations, could possibly explain the lack of observed significant effects reported in some studies. Another issue arising from the small number of studies in this area is the ability to generalise results to a broader range of athletic populations, thereby limiting definitive conclusions, as there were inconsistencies and gaps in the available literature. Unfortunately, the heterogeneity of testing methodologies and outcome measures precluded a meta-analysis to quantitatively synthesise the evidence. Consequently, we focused on providing a detailed qualitative analysis, thereby outlining potential mechanisms to inform future investigations.

## 5. Conclusions

This review identifies that greater UB strength-generating capacity may positively influence RSA by decreasing accumulated fatigue in repeated sprints. While maximal sprinting speed may also be positively affected, it is currently not clear whether shorter sprints could be supported by increased UB strength levels. Further potentially important findings from this review are the significant correlations reported between maximal trunk strength and COD performances. Moreover, trunk extensors may have important contributions to drop jump performance while also showing promise for the enhancement of other jumping variations.

## 6. Future Directions

Future cross-sectional studies should look to elucidate the relationship between maximal UB strength qualities and high-intensity running and jumping actions by conducting various strength tests for different UB muscles while accounting for the LB force-producing capacity as an influencing factor. Furthermore, given the complex interplay between the upper and lower extremities during whole-body movements, expressing UB strength relative to body mass looks crucial for accurately assessing its contribution to these actions. Training intervention studies should involve UB strength and UB power exercises aimed at maximising neuromuscular adaptations across the whole UB region. These interventions would need to standardise for the leg activation levels between the groups, ideally involving athletic populations for which high-intensity running and jumping actions are critical to competitive on-field sports performance (e.g., soccer players), with the avoidance of playing positions that prioritise UB strength for physical contacts.
